# Expression of the long noncoding RNA Neat1 in saliva, blood, and ventromedial hypothalamus of pigs exposed to psychosocial stressors

**DOI:** 10.14814/phy2.71130

**Published:** 2026-10-20

**Authors:** Dane Webster, Luoyang Ding, Song Zhang, Darryl D'Souza, Alan Tilbrook, Katelyn Tomas, Sarah Babington, Kate Plush, Dominique Blache, Archa Fox

**Affiliations:** ^1^ School of Human Sciences The University of Western Australia Crawley Western Australia Australia; ^2^ UWA School of Agriculture and Environment The University of Western Australia Crawley Western Australia Australia; ^3^ SunPork Group Eagle Farm Queensland Australia; ^4^ School of Veterinary Science The University of Queensland Brisbane Queensland Australia

**Keywords:** biomarkers, Neat1, paraspeckle, physiological stress, pig

## Abstract

The long noncoding RNA Nuclear Paraspeckle Assembly Transcript 1 (NEAT1) is a master regulator of transcriptional and posttranscriptional changes in stress responses. Here we assessed Neat1 lncRNA expression using quantitative PCR in peripheral biological fluids (saliva and blood) and a brain region that regulates stress and emotion (ventromedial hypothalamus) of 12‐week‐old pigs exposed to chronic (social mixing causing conflict) and acute (restraint) psychosocial challenges. We demonstrated that Neat1 is measurable in pig saliva, blood, and brain samples. Successful Neat1 lncRNA measurements in all three tissue types further established both broad tissue expression as well as the cross‐species conserved sequence of the *NEAT1* gene. We found that Neat1_2 and Neat1 total expression did not correlate between tissues, suggesting variable expression throughout the body, and no significant differences in Neat1 lncRNA levels with either stressor. We also observed across all treatment groups changes in Neat1_2 and cortisol levels comparing samples taken before the intervention, compared to after, indicating confounding factors may have impacted the model. Overall, this study demonstrates the feasibility of measuring Neat1 in biological fluids obtained from farm mammals, with further work required to assess Neat1 lncRNA as a molecule involved in the physiological response to psychosocial stress.

## 1 INTRODUCTION

Nuclear Paraspeckle Assembly Transcript 1 (NEAT1) is a mammalian long non‐coding RNA (lncRNA) that is involved in a range of biological functions in the context of stress, stress recovery, and disease (Fox et al., 2018; Hirose et al., 2019; Nakagawa et al., 2018). The role of NEAT1 has been extensively studied following strong stress stimuli that induced significant biological disruption to the cultured cell or the animal. For example, elevated levels of NEAT1 have been observed in cell culture post exposure to severe cell stressors such as heat shock (Lellahi et al., 2018), or in chemically‐induced stressors like sodium arsenate and MG132 treatment (An et al., 2019; Wang et al., 2018); all of which heavily impact cell homeostasis. In the disease context, *NEAT1* has been extensively studied in human patients and mouse models, such as in studies examining NEAT1 expression in cancer and sepsis (Huang et al., 2017; Yu et al., 2017). Comparatively, for more subtle stressors such as psychological stress, a *Neat1* knockout mouse behavioral study has suggested that Neat1 lncRNA is implicated in stress signaling within the brain to enable adaptive behavior in response to psychological stress (Kukharsky et al., 2020). However, there is a lack of equivalent studies in large animal models.

At the molecular level, the *NEAT1* gene produces two isoforms (Arkhipova & Drukker, 2025; Weghorst et al., 2024); a short form, NEAT1_1, and a long form, NEAT1_2. NEAT1_2 plays an essential scaffolding role in the formation of paraspeckles, dynamic nuclear condensates that are sensitive to stress (Bond & Fox, 2009; Ingram & Fox, 2024). NEAT1_1 has paraspeckle‐independent roles in metabolism and inflammation regulation (Park et al., 2021; Smith et al., 2022). Combined, both NEAT1 isoforms can govern transcriptional activity by interacting with RNAs and proteins in different ways (An et al., 2019; Bond & Fox, 2009; Choudhry et al., 2015; Fox et al., 2018; Hirose et al., 2014; Ingram & Fox, 2024; Li et al., 2017; Liu et al., 2022; Park et al., 2021; Wang et al., 2019). Thus, the broad, high order function of NEAT1 lncRNA in an array of stressors and diseases makes it a good candidate to test in a pilot as a molecule of the mammalian physiological stress response to different psychological stressors.

NEAT1 expression has been measured previously across many biological samples with RT‐qPCR. For example, in humans, NEAT1 lncRNA has been measured in liver (Wang et al., 2020), colon (Pan et al., 2021), brain regions (Cheng et al., 2018; Katsel et al., 2019), muscle (Jensen et al., 2012; Wang et al., 2019), and bone (Liu et al., 2022). Certain cell subpopulations express higher NEAT1 within tissues, suggesting particular functionality for specific cell types in response to environmental triggers (Nakagawa et al., 2011), and NEAT1 expression is absent in some cells such as stem cells until they differentiate (Chen & Carmichael, 2009). Additionally, NEAT1 has been measured in the blood of humans with pathologies such as sepsis (Huang et al., 2017), ovarian and colorectal cancer (Yu et al., 2017), and liver diseases (Bu et al., 2020). In one study, a higher NEAT1 expression in saliva samples was reported from COVID‐19 patients compared to controls (Rodrigues et al., 2021). Therefore, NEAT1 is expressed not only in cells and tissues but also in biological fluids that are easily accessible, such as blood and saliva. The possibility of using non‐invasive sampling of biological media would increase the chance of any biomarker being adopted in both the study of stress physiology and the animal industries.

In the context of a biomarker of animal welfare states for farm mammals, molecules associated with physiological responses to psychological stress are attractive candidates. Neat1 lncRNA is a well‐established molecule that is responsive to stressors. However, Neat1 lncRNA has mainly been studied in laboratory animals such as mice rather than in large mammals. Since the valence of welfare can shift from positive to negative, it is important to investigate the relevance of a molecule such as Neat1 lncRNA in biological samples obtained from animals exposed to enrichment conditions conducive to positive welfare states or exposed to psychological stressors that induce fear and negative welfare states (Mellor & Beausoleil, 2015).

To explore the relevance of Neat1 lncRNA as a possible indicator of negative or positive welfare for farm mammals, we aimed to test if the expression of Neat1 changes in response to psychological stressors and positive enrichment in *sus scrofa* (pig). In this pilot study, we test if Neat1 expression in pigs changes in response to fear and conflict, hereafter psychosocial stress, in easily obtained biological fluids of blood and saliva, as well as in the ventromedial hypothalamus, a brain region involved in the generation of emotions (Dalgleish, 2004; Silva et al., 2016). The present study was thus designed to first develop the methodology to measure the expression of both Neat1_1 and Neat1_2 isoforms (hereafter called Neat1 total), as well as Neat1_2 in pigs. The samples were obtained in an experiment investigating the impact of long‐term states, social mixing as a psychosocial stress condition or a positive enrichment condition, as well as short‐term challenges, snout snare restraint, known to induce psychosocial stress (Roozen et al., 1995; Hemsworth et al., 1986; Merlot et al., 2011; Terry et al., 2021; Tong et al., 2019; Wirthgen et al., 2017). We hypothesized that in response to either of the two psychosocial challenges, the expression of Neat1 total and Neat1_2 would increase in peripheral biological fluid (blood and saliva) and in the pig brain (ventromedial hypothalamus).

## 2 METHODS

### 2.1 Ethics

This experiment was approved by the CHM Alliance Pty Ltd. Animal Ethics Committee (PP 151/22) and ratified by The University of Queensland Animal Ethics Committee. All the animal work was carried out in accordance with the ARRIVE guidelines and the Australian Code of Practice for the Care and Use of Animals for Scientific Purposes (8th Edition, 2013).

### 2.2 Experimental animals and design

A total of 18 male and 18 female pigs, *sus scrofa* (12 weeks old) kept in a naturally ventilated shed at CHM Westbrook Research facility (Queensland, 27.60° S, 151.87° E), were used for this study. The experimental animals were part of a larger cohort of 144 pigs, housed in six groups of 24 mixed‐sex individuals in each pen (2.6 × 6.5 m) that had partially slatted and solid concrete flooring. Brain samples from the same pigs were used in another study, where this experimental design and treatments are also described (Figure 1) (Blache et al., 2024).

**FIGURE 1:**
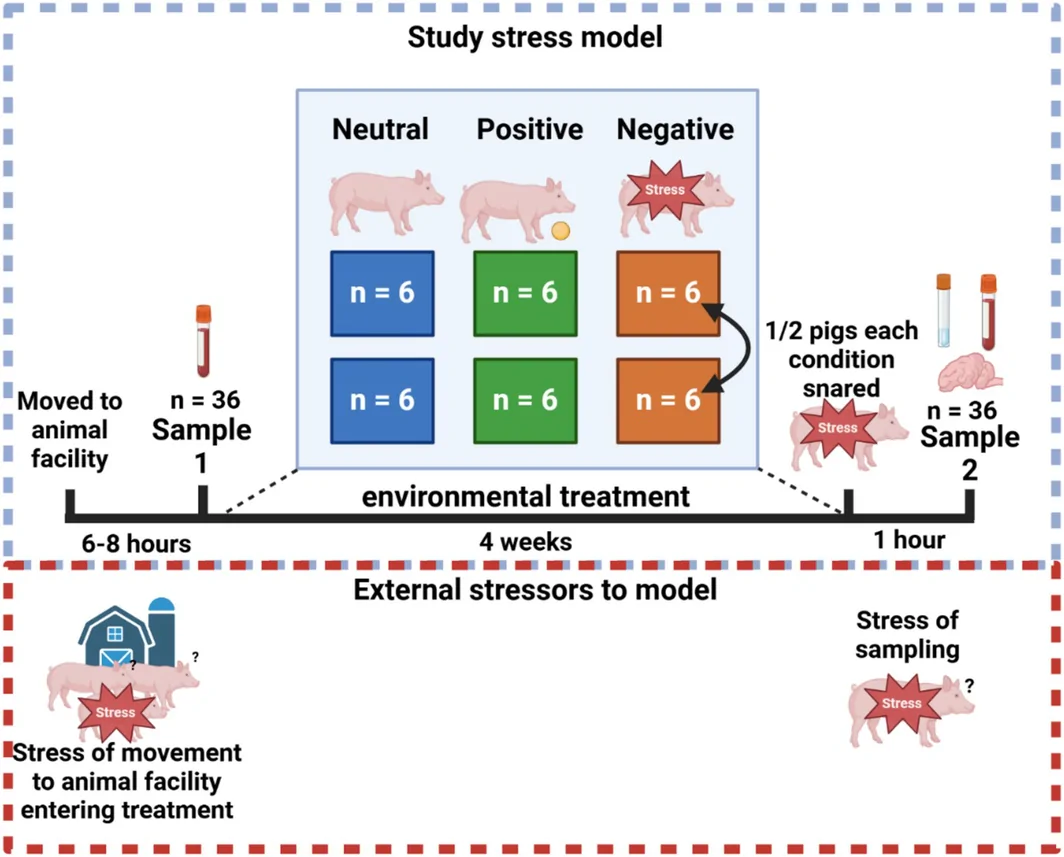
An overview of the experiment, animals and design. One hundred and forty‐four pigs were used for the entire project, with sex balanced pigs randomly assigned into three environmental treatments (Neutral, Positive, and Negative) for 4 weeks. Thirty‐six pigs were randomly selected for biological sampling, and a blood sample was taken. The stress model encompassed an environmental treatment for 4 weeks; half of the subset pigs received an aversive stimulus (snout snare restraint), and the other half did not (no snare) to stimulate an acute stress response. Other external stressors placed on the pigs included the stress of movement to the animal facility on the first morning of sampling and the stress of sampling at the end time point. One hour after stimulation, a second saliva and blood sample were collected. Pigs were then humanely killed, and different regions of the brain were dissected and snap frozen for later analysis, with the ventromedial hypothalamus used in this study.

Twelve pigs were randomly selected from each of the three different treatment groups (balanced for sex and across the six holding pens) that received the positive, the negative, and the control environmental treatments for 4 weeks. The environmental treatments were detailed as follows:

- Neutral: Normal husbandry conditions experienced by pigs in a standard conventional grower housing.
- Positive: Positive environmental treatment – normal husbandry conditions with positive stockperson interactions and enrichment (new toys every week and new hay daily), following a similar protocol to that described by Hemsworth et al. (1986).
- Negative: Negative environmental treatment–normal husbandry conditions but with regular changing of pen mates, following methodologies shown to act as an environmental psychosocial stressor described by Hemsworth (2018).

The weight of each pig was measured at the beginning and end of treatment. The average daily weight gain (ADG) was calculated as the change in weight (final weight minus starting weight) divided by the days between weight recordings.

At the end of 4‐week environmental treatment, half of the experimental pigs from each environmental treatment group were randomly selected (sex‐balanced) for an acute stress treatment with restraint via snout snare for 5 min (Terry et al., 2021).

### 2.3 Blood, saliva and ventromedial hypothalamus collection

After being moved to the experimental facility, pigs had whole blood collected from the jugular vein and stored in EDTA vacutainer collection tubes. For the measurement of gene expression, 2 mL blood was aliquoted into cryovials and snap frozen with liquid nitrogen immediately. The remainder of the blood sample left in the EDTA tube was refrigerated immediately and centrifuged to separate and collect the plasma for cortisol measurement.

At final sampling, after 1‐h post 5‐min snout snare or no snout snare, each animal was removed from its pen and held by a trained operator. The blood was collected for both gene expression and cortisol as described from the jugular vein. The timing of this last blood sampling was chosen to match the increase in cortisol usually observed after exposure to a stressor in pigs (Turner & Tilbrook, 2006). We did not repeat blood sampling to avoid extra stress due to handling and because, as described below, the brain was collected within a few minutes after the last blood sample. To collect saliva, cotton swabs were wrapped around string in which pigs chewed. The cotton swabs were then preserved in liquid nitrogen. To collect the brain, pigs were then sedated with an injection of a mixture of Zoletil (4 mg.kg^−1^, Virbac, Milperra, NSW, Australia) and Xylazine (2 mg.kg^−1^, Randlab, Milperra, NSW, Australia) in the ear vein. Once unconscious, the animal was injected intravenously into a jugular vein with an overdose of pentobarbital (140 mg.kg^−1^, Virbac, Milperra, NSW, Australia). A veterinarian confirmed death by the absence of cardiac activity. The head was then severed using a sharp knife, the skin on the top of the skull was removed, and the skull was opened using a bone saw (Hebu, Tek equipment, Preston, VIC, Australia). Within 15 min of confirmation of being deceased, the ventromedial hypothalamus was collected and snap frozen in liquid nitrogen.

### 2.4 Measurement of plasma cortisol

Plasma cortisol was quantified on the day of collection. The plasma concentration of cortisol was measured in duplicate using the manufacturer's protocol and reagents from the MP Biomedical I125 RIA cortisol Kit (#07‐221106, Australian Bioresearch, Perth). The limit of detection was 0.25 ng.mL^−1^ and the intra‐assay variability was 6.5% (1.4 ng.mL^−1^) and 1.8% (3.2 ng.mL^−1^).

### 2.5 Quantitative polymerase chain reaction (qPCR)

Total RNA from the blood, saliva, and ventromedial hypothalamus samples was extracted using Mini NucleoSpin® RNA Blood kit (740200.50), Monarch Total RNA Miniprep Kit (NEB #T2010), and QIAzol lysis reagent (79306), respectively.

Genomic DNA was removed prior to reverse transcription for ventromedial hypothalamus and saliva RNA samples using gDNA Wipeout from the Quantitect reverse transcription kit as per recommendations. For blood, gDNA was removed from total RNA using Deoxyribonuclease (DNase) treatment (M610A, Promega). The Quantitect Reverse Transcription kit by Qiagen (205314) was used to produce complementary deoxyribonucleic acid (cDNA) from RNA. After cDNA synthesis, concentrated samples were diluted with water for ventromedial hypothalamus samples (1 cDNA:5 H_2_O), blood samples (1:3) and saliva samples (1:2.33) and stored at −20°C prior to qPCR analysis.

Real‐time qPCR was completed using the QuantiNova SYBR Green PCR Kit (Qiagen, 208,252) on the Rotor‐Gene Q (QIAGEN). Relative gene expression was calculated using Pfaffl (Pfaffl, 2001). For choice of suitable reference genes for each tissue, the raw expression data was analyzed using the algorithms of geNorm, NormFinder, comparative delta Ct method, and BestKeeper (Andersen et al., 2004; Pfaffl et al., 2004; Silver et al., 2006; Vandesompele et al., 2002). RefFinder software was then used to integrate the results from the four algorithms and provide a comprehensive ranking for the most stable candidate reference genes used for normalization (Xie et al., 2012). Rpl4 and Gapdh were used for normalization in pig ventromedial hypothalamus, Ppia and Gapdh for pig saliva, and Ppia and Rpl4 for pig blood. The geometric mean of these housekeeping genes that were found to be most stable in respective tissues was used to calibrate Neat1 total and Neat1_2 expression data. Percentage of Neat1_2 was calculated from dividing Neat1_2 expression by Neat1 total.

### 2.6 Selection of reference genes and primer design for Neat1 qPCR

The use of the geometric mean of Rpl4 and Ppia genes has been recommended for a suitable normalization in pig samples (Cinar et al., 2013). In addition, Gapdh was selected as a third reference gene (Table 1) (Kozera & Rapacz, 2013). The NCBI GenBank Primer‐BLAST tool was used to design primers or gathered from prior study (Cinar et al., 2013; NCBI, 2023). The pig *ss_Neat1* locus and full transcript sequences were not annotated at the time of the study; therefore we used GeneBank BLAST to identify a locus that had ~60% similarity with human *NEAT1* (NCBI, 2024). Highly conserved sequences between the candidate pig *ss_Neat1* region and human *NEAT1* gene were chosen to produce multiple Neat1_2 and Neat1 total primer targets (Figure 3A). The most suitable primer target for each of Neat1 total and Neat1_2 was chosen, based on RT‐qPCR melt curve analysis, sequencing verification of amplicon and amplification efficiency.

**TABLE 1:** qPCR primer sequences. RPL4, GAPDH, and PPIA were selected as reference genes.

Gene RNA name	Gene ID (accession)	Primer sequence (5′ → 3′)	Amplicon length (bp)	Annealing temperature (°C)
Rpl4	XM_005659862	F: AGGAGGCTGTTCTGCTTCTG R: TCCAGGGATGTTTCTGAAGG	185	55.6°C
Gapdh	NM_001206359	F: TCGGAGTGAACGGATTTGG R: GTGGAGGTCAATGAAGGGGTC	107	57.6°C
Ppia	NM_214353	F: CACAAACGGTTCCCAGTTTT R: TGTCCACAGTCAGCAATGGT	171	55.6°C
Neat1 Total	‐	F: CTGTTCGGGGGTGGAGGTTT R: ACCAGGCATCACTCTGCGTT	145	60°C
Neat1_2	‐	F: CGTTGCGCTTACACTGTGGG R: ACATGCTCAGTGACCAGGCA	186	60°C

### 2.7 Statistical analysis

IBM SPSS Version 29.0 was used for all statistics of repeated measure general mixed models and ANOVA. Normality was assessed using Shapiro–Wilk Test and Levene's Test, data showing non‐normality were transformed where possible. When normality through transformation was not achieved, non‐parametric data was analyzed through Kruskal‐Wallis H Test. Due to non‐normality of Neat1 total in the first sample, Neat1 total was analyzed using change in Neat1 total from first to second sampling. All results are expressed as mean ± standard deviation unless otherwise specified. Covariates were pig sex and individual pigs. When main effects or interaction were found for sex, the variate was made a fixed factor. Pen was analyzed as a random factor, with the fixed factors environmental and acute treatments statistically nested in the model within pen variate. GraphPad Prism 8.4 was used for all correlation statistical analysis. End time point samples were used for all correlation statistical analysis since all tissues were collected at this point and no differences were found between treatment groups. A Pearson's correlation coefficient was first computed to confirm linearity, followed by simple linear regression to assess the linear relationship between variables. The *p* value is reported from simple linear regression. GraphPad Prism 8.4 was used to generate all figures.

## 3 RESULTS

### 3.1 The effect of long‐term experiential conditions on average daily weight gain

In the 36 pigs that were used for biological sampling, we found no effect of environmental treatment or sex on ADG (Figure 2, *p* = 0.10 and *p* = 0.40, respectively).

**FIGURE 2:**
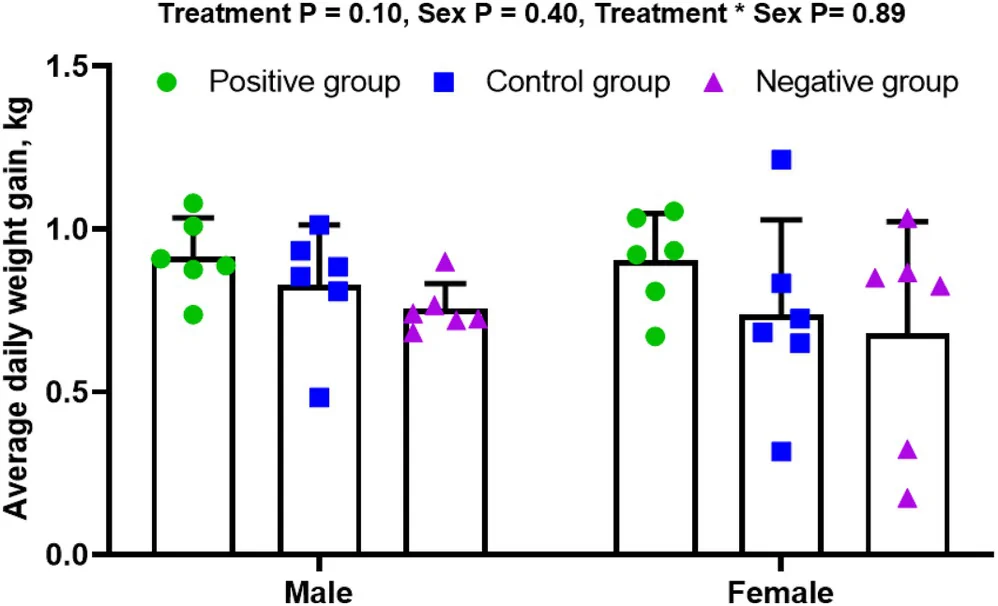
4‐week ADG of pigs exposed to conditions of normal husbandry (neutral), environmental enrichment (positive), and weekly pen mixing (negative). ADG was calculated from 36 pigs that were used for biological sample collection. Data are shown as mean ± SD.

### 3.2 The effect of stress challenges on Neat1 expression at end time point sampling in the ventromedial hypothalamus and saliva

RNA extracted from each tissue produced variable yields (Supplementary S1). On average, ventromedial hypothalamus (63.69 ± 16.45 mg) had the highest RNA yield of 34.36 ± 14.35 μg, with a 260/280 ratio of 2.00 ± 0.006 and 260/230 of 2.06 ± 0.20. Whole blood (using 400 μL of starting sample) yielded 6.10 ± 2.39 μg of RNA, with a 260/280 ratio of 2.05 ± 0.004 and 260/230 ratio of 1.87 ± 0.20. Saliva (2 cotton swabs per sample from the same pig) yielded the least RNA, with an output of 1.86 ± 0.84 μg, with a 260/280 ratio of 1.58 ± 0.11 and 260/230 ratio of 0.81 ± 0.18.

Following RT‐qPCR analysis, we found there was no effect of environmental or acute treatment on Neat1 total expression in pig ventromedial hypothalamus (*p* = 0.171 and *p* = 0.272, respectively, Figure 3B). There was also no effect of environmental or acute treatment on pig ventromedial hypothalamus Neat1_2 expression (*p* = 0.293 and *p* = 0.517, respectively, Figure 3C). The percentage of Neat1_2 represents the change in isoform between Neat1_1 and Neat1_2, with no effect of environmental or acute treatment on ventromedial hypothalamus percentage of Neat1_2 expression being found (*p* = 0.995 and *p* = 0.762, respectively).

**FIGURE 3:**
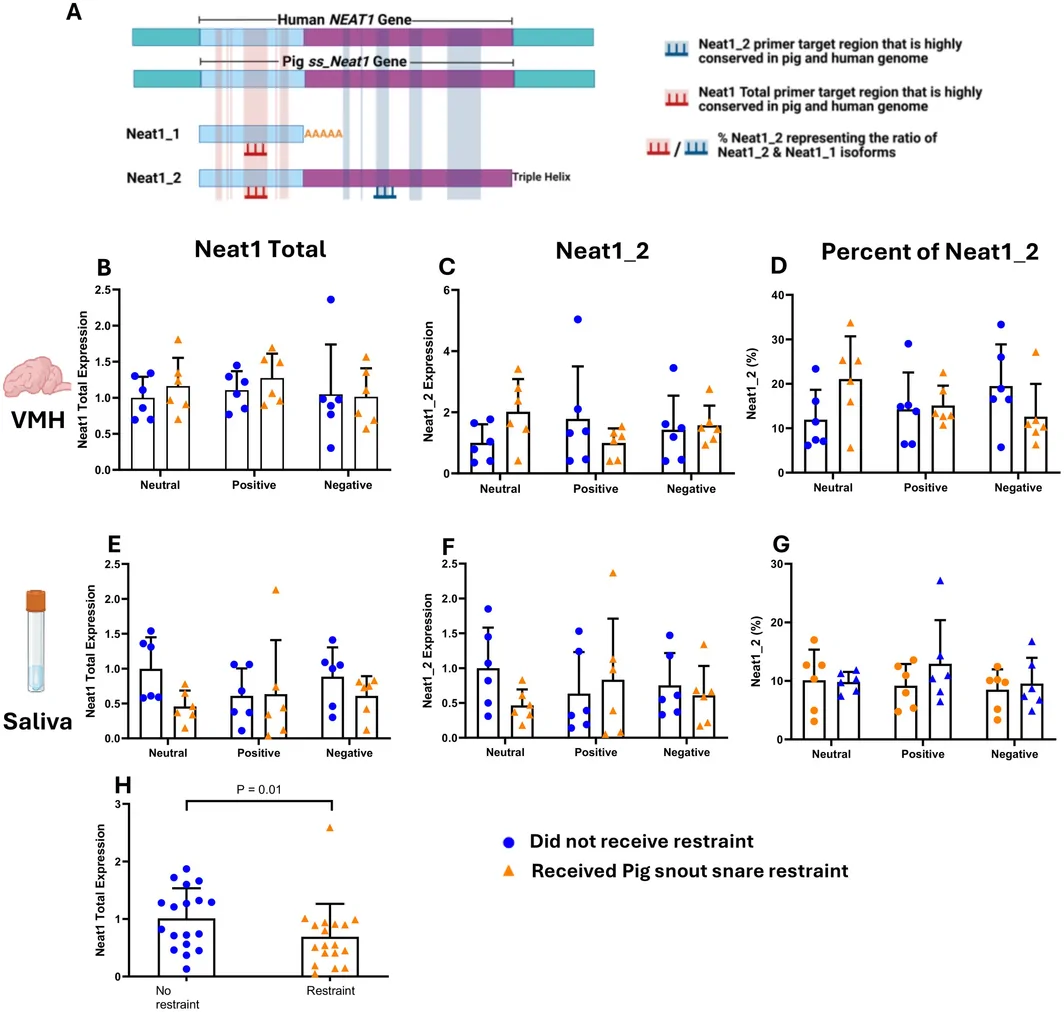
Neat1 expression in the pig ventromedial hypothalamus (VMH) and pig saliva in response to environmental and snare stressors. (A) Targeting of the pig Neat1 orthologs in RT‐qPCR. (B–D) Expression of Neat1 total (A), Neat1_2 (B), and percentage of Neat1_2 (C) in pig ventromedial hypothalamus. (E–G) Expression of Neat1 total (D), Neat1_2 (E), and percentage of Neat1_2 (F) in pig saliva. (H) Comparison of pigs who received a snare and did not receive a snare on effect of Neat1 total expression in pig saliva. Graphs are presented as individual pigs with mean ± SD (panels B–H).

We next analyzed the saliva, observing no effect of environmental treatment on the expression of Neat1 total (*p* = 0.15, Figure 3E). However, the pigs that received acute stress had significantly lower expression of Neat1 total compared to pigs that received no acute stress (*p* = 0.01, Figure 3H). There was no effect of environmental stress or acute stress on the expression of Neat1_2 (*p* = 0.90 and *p* = 0.54, respectively, Figure 3F) or the percentage of Neat1_2 (*p* = 0.73 and *p* = 0.53, respectively, Figure 3G) in the saliva of experimental pigs.

### 3.3 The effect of challenges in pig blood on Neat1 expression and plasma cortisol at two time points

We next examined Neat1 expression in blood. There was no effect of environmental or acute treatment on change in Neat1 total expression in pig blood (*p* = 0.42 and *p* = 0.06 respectively, Figure 4A). There were no effects of environmental treatment or acute treatment on the Neat1_2 expression in pig blood (*p* = 0.90 and *p* = 0.81 respectively, Figure 4B). However, there was an effect of time (*p* = 0.023), where Neat1_2 expression was higher at the first sampling (1.40 ± 1.09) in comparison to the end of treatment (0.32 ± 0.24, Figure 4B). The percentage of Neat1_2 in the blood was not different over time (*p* = 0.07, Figure 4C), however, the trend suggests that a reaction to a stress stimulus may result in a change of Neat1 isoform. In regard to the stress model treatments, there was no effect of environmental or acute treatment on pig blood percentage of Neat1_2 expression (*p* = 0.65 and *p* = 0.19 respectively, Figure 4A–C).

**FIGURE 4:**
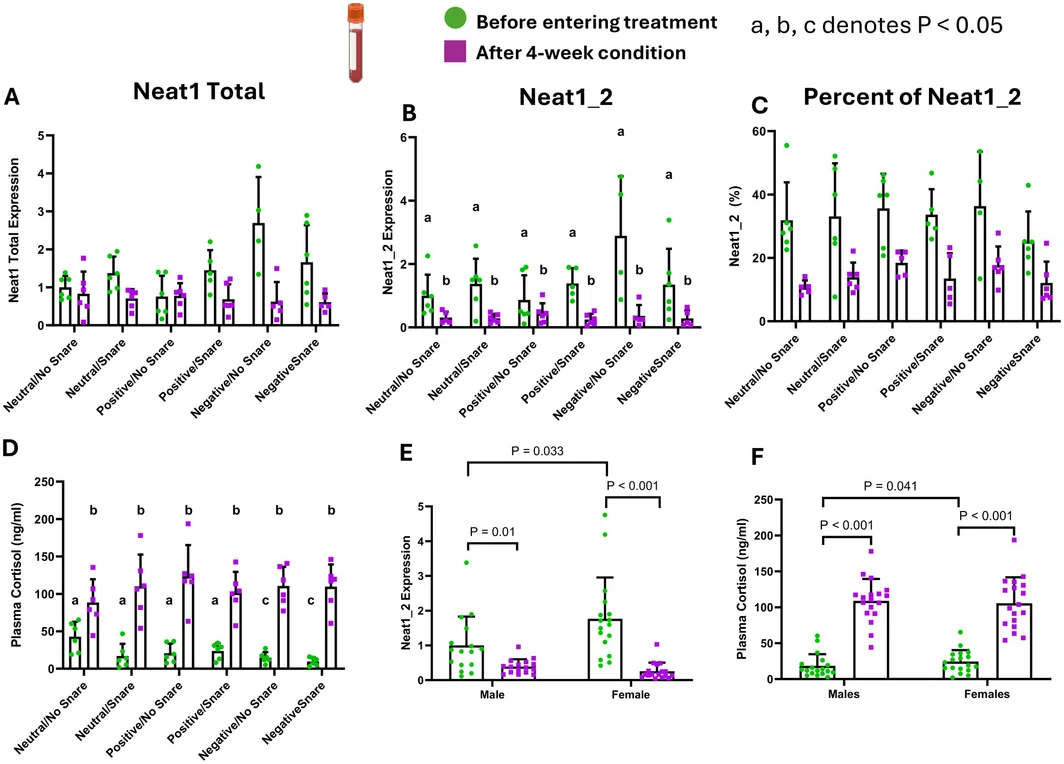
Neat1 expression and cortisol levels in pig blood in response to environmental and snare stressors. (A–C) Neat1 total expression (A), Neat1_2 expression (B), and percentage of Neat1_2 (C) before and after environmental condition and aversive snare treatment. (D) Neat1_2 expression in male and female pigs before and after environmental condition. (E) Plasma cortisol before and after 4‐weeks in environmental treatment and aversive snare treatment. (F) Plasma cortisol before entering treatment and after 4‐weeks by sex. The different superscripts suggest a significant difference, *p* < 0.05. Graphs are presented as individual pigs with mean ± SD.

An interaction between time of sampling and sex interaction on Neat1_2 expression in blood was found (*p* = 0.025). Before entering the treatments, female pigs (1.33 ± 0.90) had higher NEAT1_2 expression than male pigs (0.76 ± 0.63) (*p* = 0.033, Bonferroni pairwise analysis). In comparison, Neat1 expression decreased in both female (0.19 ± 0.19) and male (0.29 ± 0.16) pigs at the second biological sampling point compared to 4‐week prior baseline (*p* < 0.001 and *p* = 0.010 respectively, Bonferroni pairwise analysis, Figure 4E,F).

There was an interaction between time of sampling and environmental treatment on the concentration of cortisol in the plasma (*p* = 0.038, Figure 4D). The baseline samples taken before the start of environmental treatment showed a trend towards a difference between groups (*p* = 0.054, Figure 4D), with pigs that were to be placed in the positive enrichment (22.19 ± 11.26 ng/mL) and the pigs that were to be placed in normal husbandry conditions (29.90 ± 21.99 ng/mL) having higher cortisol levels than the pigs that were to be placed in the negative treatment (12.21 ± 6.70 ng/mL) (*p* = 0.06 and *p* = 0.06 respectively, Bonferroni pairwise analysis, Figure 4D). At the end of the environmental treatment, the cortisol concentrations were higher in all of the groups compared to the levels measured prior to treatment, with the normal husbandry conditions (99.33 ± 37.22 ng/mL), positive enrichment (112.57 ± 35.65 ng/mL) and negative treatment (110.07 ± 26.44 ng/mL), all being significantly higher than their respective baseline levels (*p* < 0.001, Bonferroni pairwise analysis, Figure 4D). Acute stress had no effect on the concentration of plasma cortisol (*p* = 0.14). However, the concentration of cortisol was higher at the second sampling than it was at the baseline (*p* < 0.001).

There was an interaction between time of sampling and sex on plasma cortisol (*p* = 0.046). Before entering the treatments, the female pigs (24.37 ± 15.92 ng/mL) had a higher plasma concentration of cortisol than did the male pigs (18.50 ± 16.22 ng/mL) (*p* = 0.041, Bonferroni pairwise analysis). In both the female (105.65 ± 36.15 ng/mL) and male (108.99 ± 30.46 ng/mL) pigs, the plasma cortisol level was elevated at the second sampling (*p* < 0.001 for both, Figure 4F).

At the start of the current study, the concentration of cortisol in plasma was not different between the groups in the different environmental treatments (*p* = 0.054) or between the groups that were later subjected, or not, to acute stress (*p* = 0.14). There was no interaction between environmental treatment and acute stress (*p* = 0.52). At the end of the current study, there was again no significant effect of environmental treatment (*p* = 0.08) or acute stress (*p* = 0.66) on the concentration of cortisol in plasma. Similarly, there was no interaction between environmental treatment and acute stress (*p* = 0.45).

### 3.4 Correlations of Neat1 expression in blood, saliva and ventromedial hypothalamus at end time point sampling

We were interested if Neat1 levels correlated within different tissues in the same individual animal. Specifically, we were interested if levels of Neat1 in the easily obtainable fluids of blood and saliva correlated with those measured in the ventromedial hypothalamus. Hence, we next examined if there was tissue expression correlation for Neat1_2, Neat1 total, and the percentage of Neat1_2 expression in the ventromedial hypothalamus, blood, and saliva for each individual pig using samples from the end time point sampling only. We did not find any correlation in Neat1 total expression (*R* ^2^ = 0.015, *p* = 0.49), Neat1_2 expression (*R* ^2^ = 0.011, *p* = 0.57), or percentage of Neat1_2 (*R* ^2^ = 0.003, *p* = 0.77) (Figure 5a–c). There was also no evidence of a correlation for Neat1 total expression (*R* ^2^ = 0.002, *p* = 0.82), Neat1_2 expression (*R* ^2^ = 0.01, *p* = 0.49), or percentage of Neat1_2 (*R* ^2^ = 0.10, *p* = 0.06) between the saliva and ventromedial hypothalamus (Figure 5d–f). Lastly, no evidence of a correlation in Neat1 total expression (*R* ^2^ = 0.05, *p* = 0.20), Neat1_2 expression (*R* ^2^ = 0.01, *p* = 0.51), or percentage of Neat1_2 (*R* ^2^ = 0.003, *p* = 0.75) between the saliva and the blood was found (Figure 5g–i).

**FIGURE 5:**
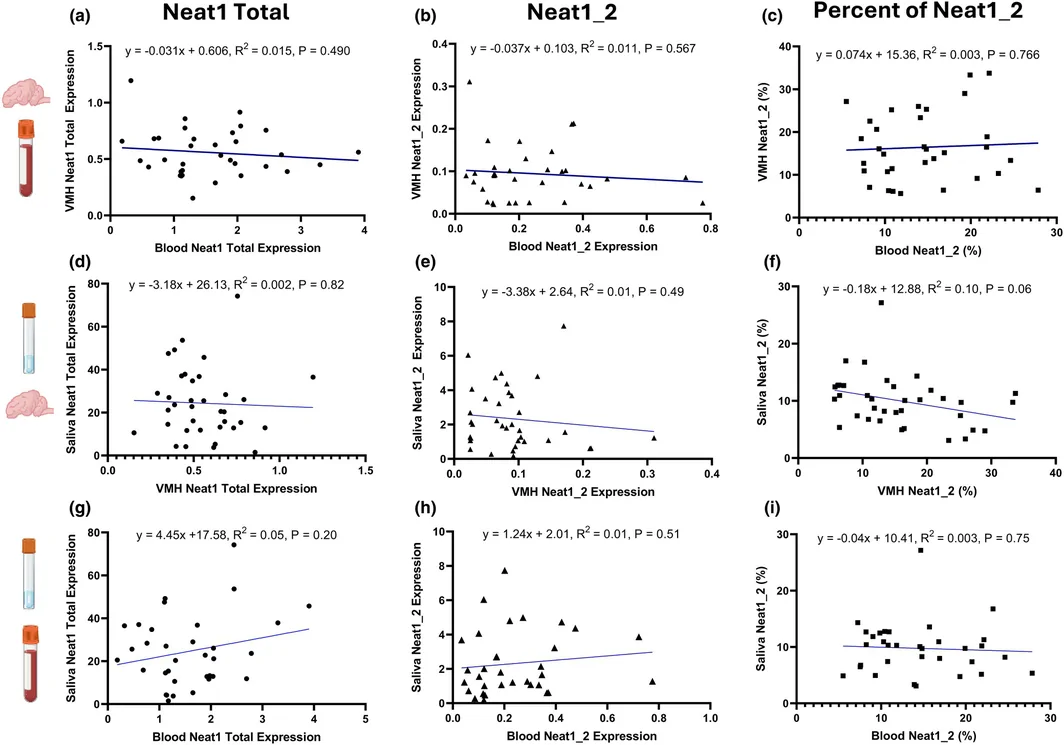
Correlations of Neat1 expression in the ventromedial hypothalamus, blood, and saliva. (a–c) Correlation of Neat1 total (a), Neat1_2 (b), and the percentage of Neat1_2 (c) in the ventromedial hypothalamus and blood. (d–f) Correlation of Neat1 total (d), Neat1_2 (e), and the percentage of Neat1_2 (f) in the saliva and ventromedial hypothalamus. (g–i): Correlation of Neat1 total (g), Neat1_2 (h), and the percentage of Neat1_2 (i) in the saliva and blood.

### 3.5 Correlations of Neat1 expression in blood, saliva and ventromedial hypothalamus with plasma cortisol at end time point sampling

Although the timing and response of Neat1 production can be highly variable, as well as the mechanisms of cortisol and Neat1 being different, we also calculated correlations of cortisol secretion and Neat1 expression at the end time point sampling. As expected, we did not observe any correlation between plasma cortisol and Neat1 total expression (*R* ^2^ = 0.001, *p* = 0.88), Neat1_2 expression (*R* ^2^ = 0.018, *p* = 0.44) or percentage of Neat1_2 (*R* ^2^ = 0.016, *p* = 0.47) in the ventromedial hypothalamus (Figure 6a–c). We also did not find any correlation in pig blood with cortisol secretion and Neat1 total expression (*R* ^2^ = 0.035, *p* = 0.29), Neat1_2 expression (*R* ^2^ = 0.138, *p* = 0.07) or percentage of Neat1_2 (*R* ^2^ = 0.009, *p* = 0.59) (Figure 6d–f). There was also no correlation with plasma cortisol secretion and Neat1 total expression (*R* ^2^ = 0.06, *p* = 0.13), Neat1_2 expression (*R* ^2^ = 0.02, *p* = 0.36) or percentage of Neat1_2 (*R* ^2^ < 0.001, *p* = 0.95) in pig saliva (Figure 6g–i).

**FIGURE 6:**
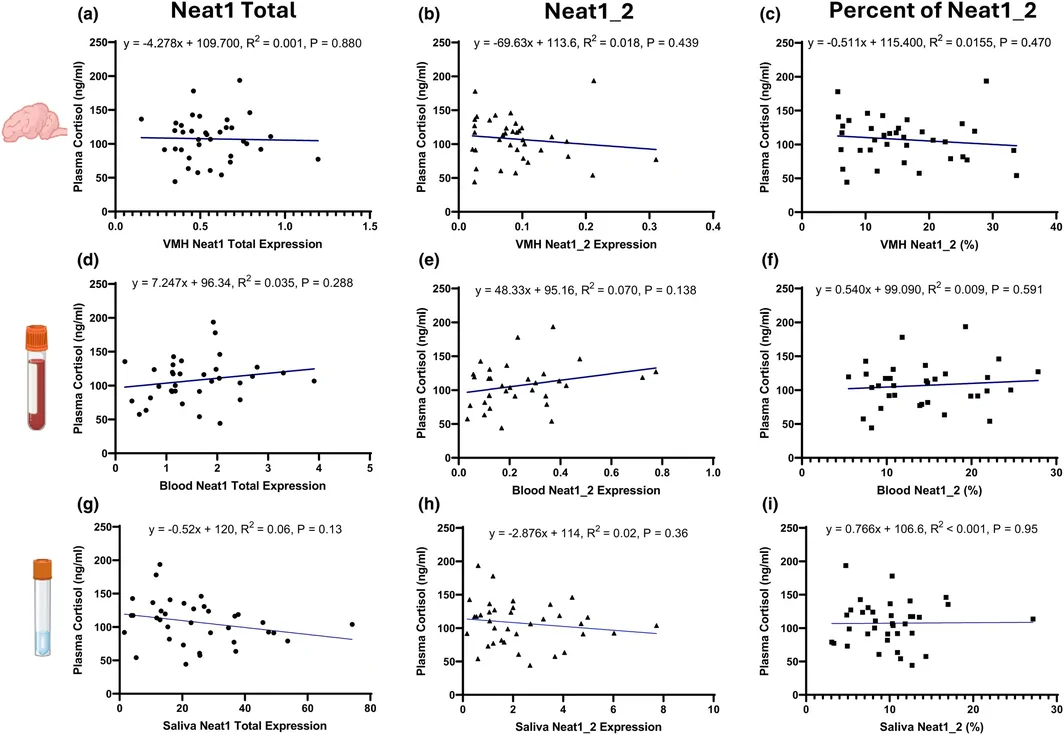
Correlations of plasma cortisol with Neat1 expression in the ventromedial hypothalamus, blood, and saliva. (a–c) Correlation of Neat1 total (a), Neat1_2 (b), and the percentage of Neat1_2 (c) in the pig ventromedial hypothalamus with plasma cortisol. (d–f) Correlation of Neat1 total (d), Neat1_2 (e), and the percentage of Neat1_2 (f) in pig blood with plasma cortisol. (g–i) Correlation of Neat1 total (g), Neat1_2 (h), and the percentage of Neat1_2 (i) in the pig saliva with plasma cortisol.

## 4 DISCUSSION

In this study, we report the measurement of the lncRNA Neat1 for the first time in the blood, saliva, and ventromedial hypothalamus of a farm mammal, the pig, using RT‐qPCR. We found Neat1 was measurable and expressed widely, confirming the wide tissue distribution of Neat1 and its conserved sequence in other species than mice and humans. Our results demonstrate that both short and long isoforms of Neat1 can be measured in the blood, saliva, and brain tissue of a large mammal. We did not find differences in Neat1 between the treatment groups; however, the expression of Neat1_2 was altered at two different exposures to two independent challenges in blood (start sampling comparison to end time point sampling). To further explore the potential of Neat1_2 as a molecule of the physiological stress response to psychosocial stress and a possible biomarker of animal welfare, it would be necessary to overcome some of the limitations of the present study, such as better control of the intensity and context of the stressors as well as the timing of the sampling in relation to the exposure to the stressors.

To our knowledge, ours is the first study to measure the expression of Neat1 in pig blood, brain, and saliva, with one prior study measuring Neat1 in pig leg muscle (Jensen et al., 2012). The methodologies used to obtain the data presented here further illustrate that the sequence for NEAT1 is well conserved amongst mammals within three additional different tissue types now added to the list of Neat1 positive samples in pig, as well as muscle (Jensen et al., 2012). Recent studies suggest that *Neat1* may also be present in certain amphibians and reptile species, as well as mammals (Weghorst et al., 2024). Due to a lack of annotation of the *ss_Neat1* gene in the pig genome bank, we could not design primers using a known *ss_Neat1* pig sequence. Hence, we designed Neat1 total and Neat1_2 primers for *sus scrofa* by aligning human *NEAT1* to the pig genome, identifying a locus the length of human NEAT1_2 with approximately 60% similarity (UCSC, 2023). From this predicted pig *ss_Neat1* locus, we identified a short span of sequence with highly homologous portions to the known human *NEAT1* sequence matched close to the 5′ prime end (as a Neat1 total primer), as well as a matched mid‐to‐end sequence (as a Neat1_2 primer) and successfully obtained amplicons. Hence, this technique may also be used for other novel genes in species hypothesized to have highly homologous sequences to a well sequenced genome (such as human, mice, or fruit fly). It should be noted that a new study has recently annotated the pig *ss_Neat1* gene, confirming our identified region (Arkhipova & Drukker, 2025).

It is interesting to consider the cellular source of Neat1 molecules in our samples. In this study we used pig blood for RNA isolation and did not first enrich for any particular cell type. However, we hypothesize the Neat1 measured in pig blood originates predominantly from the peripheral blood mononuclear cells (PBMCs). This is because a small number of studies have previously measured NEAT1 in PBMCs isolated from human blood in patients suffering sepsis (Huang et al., 2017), ovarian and colorectal cancer (Yu et al., 2017), and liver diseases (Bu et al., 2020). Interestingly, PBMCs are also present in saliva, albeit at lower levels than in blood (Vidović et al., 2012). For the source of Neat1 in the ventromedial hypothalamus, a previous study examining RNAscope® in situ hybridisation of wildtype mice brains found that the highest expression of Neat1 is in glial cells (chiefly astrocytes and oligodendrocytes) with lower expression in neurons (Kukharsky et al., 2020).

We found that a given pig with high expression in one tissue does not necessarily have high Neat1 across all tissues. This lack of correlation within different body parts within a respective pig could be representative of tissue‐specific stress responsive pathways eliciting variable Neat1 expression under subtle stress. Therefore, it would be useful to repeat the study with a more potent stressor to determine if Neat1 expression may become concordant in any individual across all biological samples under strong stress conditions. The lack of correlation between saliva Neat1 lncRNA or blood Neat1 lncRNA and hypothalamic Neat1 lncRNA may weaken our initial hypothesis that the emotional processing of psychosocial fear by the brain leads to a change in expression of systemic Neat1 lncRNA. However, the biological response to psychological stressors is complex and can involve signals that originate from peripheral organs, such as adrenal response, or from other brain regions than the hypothalamus, such as the prefrontal cortex.

The lack of impact of the two challenges on *ss_Neat1* expression could be explained by the context or the intensity of the stress induced by these challenges. In our study, we used two psychosocial challenges. Firstly, pen mixing was chosen because it was a validated negative long‐term stressor that disrupts the pigs' social hierarchies and elicits conflict and pen mate fear (Hemsworth, 2018). Pen mixing was chosen as an environmental stressor that is able to induce significant stress in pigs due to pen mate unfamiliarity (resulting in constant reestablishment of group dynamics and aggression) (Hemsworth, 2018). In addition, as a validated acute stressor, on the final day of the study, selected pigs were exposed to a snout snare as a psychosocial stressor eliciting fear (Terry et al., 2021). Though snout snares do induce a stress response, it is not a strong and durable stress response of which is consistent with our results (Terry et al., 2021). In contrast to the two stresses used in our study, the expression of NEAT1 has previously been studied in severe stressors where the cost of the stress is significant to biological health (An et al., 2019; Bu et al., 2020; Huang et al., 2017; Lellahi et al., 2018; Liu et al., 2022; Rodrigues et al., 2021; Wang et al., 2018; Yu et al., 2017). Of note, we did not detect significant differences in weight gain between pigs in any condition, suggesting the pen‐mixing model was not able to induce the desired stress response. Though a p‐value of 0.10 and the direction of weight gain, an increase in daily weight gain with enrichment and a reduction with pen‐mixing, indicated a trend.

Although the two different challenges did not impact the expression of Neat1, in contrast, Neat1_2 had an inverse change in expression pattern before chronic treatment and after, compared to that of the most common stress biomarker, plasma cortisol. Neat1_2 as a lncRNA scaffold for paraspeckle formation may suggest a role of paraspeckles in this change (Bond & Fox, 2009; Ingram & Fox, 2024). The first measurement was taken when the pigs were first placed into the experimental groups, with the second time point occurring just before final tissue harvesting. At the first time point, prior to exposure to any stressor, the expression of Neat1_2 was relatively high, while the cortisol levels (21.4 ± 16.1 ng/mL, mean ± SD) were comparable to basal levels (32.1 ± 8.8 ng/mL, mean ± SD) previously reported in growing pigs (Bakari et al., 2018), suggesting that the pigs were not experiencing a stress response. By contrast, at final tissue harvesting, Neat1_2 expression was relatively low, and plasma cortisol was high. Plasma cortisol was uniformly elevated in the second sample (107.3 ± 33.0 ng/mL, mean ± SD), suggesting that all 36 pigs were stressed on the final sample collection measurement. We suspect that the commotion caused by eliciting a snout snare on half the pigs and moving pigs out of their communal pens for the final blood sample and the procedure of human killing would have likely induced a strong stress response in all the pigs, possibly masking the effect of both the acute challenges and the chronic challenge in the prior weeks.

The time effect of Neat1_2 reduction in blood could be due to aging. Pigs were kept for 4 weeks between first and second sampling, equivalent to comparison of a 12‐week‐old pig in paired blood sample to a 16‐week‐old pig. We speculate that the reduction in Neat1_2 is not age related due to the difference in age being modest. In a pig's life cycle, this 1‐month difference is comparative to a late nursery/weaner stage (12‐week‐old pig) to an early‐mid grower pig (16‐week‐old). The pigs have also not yet reached sexual maturity by the 16‐week‐old age (Patterson et al., 2010). In NEAT1 studies, there is evidence that there is differential expression of NEAT1 in aging as well as in age related diseases; however, these age differences are equivalent to 20+ year differences in humans (Wang et al., 2025; Zhang et al., 2022), 14+ month differences in mice (Butler et al., 2019; Wang et al., 2025; Zhang et al., 2022), or older‐aging disease cohorts such as Alzheimer's (Irwin et al., 2023; Puthiyedth et al., 2016). To note, we did not measure age‐related markers, so age cannot be excluded, and the absence of an effect of age is speculative.

It should be noted that the nature of our study restricted the number and timing of sampling. This is important as the timing of sample collection after exposure to the stressor may be important for detecting Neat1 changes. Cell culture studies have shown an increase in Neat1_2 expression 4–12 h after stress exposure, sometimes as long as 24 h post‐stress, such as in response to proteasome inhibition treatment (Hirose et al., 2014), as well as oxidative stress treatment (An et al., 2019). It is hypothesized that several upstream changes are required for changes in Neat1_2 expression, such as stress granule formation (An et al., 2019), and consequently, an extended time course post‐stress (6–12 or even 24 h post‐stress) is required to observe elevated Neat1_2 expression in cell‐stress models (An et al., 2019; Lellahi et al., 2018; Liu et al., 2022; Wang et al., 2018). Hence, the timing of the first sample collection being 2–4 h after the animals were transferred from their normal nursery to the experimental nursery showed relatively high Neat1_2 levels. Therefore, one possible explanation is that Neat1_2 levels were increased for only 2–4 h after transfer. Without repeated sampling on the first day, the existence of a transient increase followed by a return to baseline levels remains speculative. In contrast, our 1‐h time post‐restraint (acute stress) sample collection may have been collected too soon to measure an increase in Neat1. We selected a one‐hour time point as an intermediary window because the dynamics of the response of NEAT1 to psychosocial stress were unknown, and repeated blood sampling is known to cause a stress response in pigs (Roozen et al., 1995; Merlot et al., 2011; Wirthgen et al., 2017).

The original design of the experiment aimed to contrast between a positively perceived environment with the addition of play toys and patting, and a negatively perceived environment because of the pen‐mixing. However, the design failed to induce this contrast since the average daily weight gain did not decrease with the long‐term pen‐mixing, contrary to previous reports (Hemsworth, 2018). To best investigate these phenomena, we suggest future experimental designs consider contrasting life situations that are perceived either positively or negatively by the animals with more extensive controls. We also suggest that studying acute or chronic stress in isolation rather than a mixed model could also be done to reduce confounding variables. More blood samples could also be taken in the weeks leading up to final sampling as well as before elicitation of acute stress. This would have enabled greater insight into the effects of both our acute and environment treatments, as well as confounding effects.

Despite the study's limitations, the present study demonstrates that the level of expression of Neat1 is affected by two independent pig experiences detectable in blood by measurement at two different collection times. The lack of repeated sampling and the limited context behind the stressors used in the present study did not allow us to conclude that the observed changes in Neat1_2 expression are true biomarkers of the response to psychosocial stress. The present study was an exploratory study aiming to demonstrate that Neat1 lncRNA can be measured in pig blood and saliva, and that NEAT 1 expression is somewhat sensitive to some kind of emotional experience. Therefore, the relevance of the expression of Neat1 should be further tested in pigs using different stressful events, such as handling techniques, auditory overstimulation, isolation, or disruption of social bonds. In addition, a study of the expression of Neat1 in other regions of the brain involved in emotions, such as the prefrontal cortex, would clarify the role of central expression of Neat1 in the response to stress. Lastly, since ncRNAs are conserved between mammalian species, the expression of Neat1 in response to exposure to stressors could be investigated in other domesticated or non‐domesticated species.

## AUTHOR CONTRIBUTIONS

**Dane Webster:** Data curation; formal analysis; investigation; methodology. **Luoyang Ding:** Data curation; formal analysis; supervision. **Song Zhang:** Formal analysis; investigation; methodology; supervision. **Darryl D'Souza:** Conceptualization; data curation; funding acquisition. **Alan Tilbrook:** Conceptualization; funding acquisition; methodology; project administration. **Katelyn Tomas:** Conceptualization; data curation; investigation. **Sarah Babington:** Data curation; formal analysis; investigation; methodology. **Kate Plush:** Conceptualization; funding acquisition; investigation. **Dominique Blache:** Conceptualization; data curation; funding acquisition; project administration; supervision. **Archa Fox:** Conceptualization; funding acquisition; investigation; project administration; supervision.

## FUNDING INFORMATION

This work was funded by the Australasian Pork Research Institute Ltd. (Project 5A‐113), The University of Queensland, and The University of Western Australia.

## CONFLICT OF INTEREST STATEMENT

K.P. and D.D. are employees of SunPork which is one of the main pig producers in Australia. Their contributions are described in author contributions. The other authors declare no conflict of interest.

## Supporting information


Data S1.

## Data Availability

The datasets used and/or analyzed during the current study are available from the corresponding author on reasonable request.

## References

[phy271130-bib-0001] An, H. , Tan, J. T. , & Shelkovnikova, T. A. (2019). Stress granules regulate stress‐induced paraspeckle assembly. The Journal of Cell Biology, 218, 4127–4140. 10.1083/jcb.201904098 PMC6891081 31636118

[phy271130-bib-0002] Andersen, C. L. , Jensen, J. L. , & Ørntoft, T. F. (2004). Normalization of real‐time quantitative reverse transcription‐PCR data: A model‐based variance estimation approach to identify genes suited for normalization, applied to bladder and colon cancer data sets. Cancer Research, 64, 5245–5250. 10.1158/0008-5472.CAN-04-0496 15289330

[phy271130-bib-0003] Arkhipova, K. , & Drukker, M. (2025). Phylogenetic analysis of NEAT1 and MALAT1 long non‐coding RNAs highlights structure–function relationships in Paraspeckle biology. Molecular Biology and Evolution, 43, msaf265. 10.1093/molbev/msaf265 PMC12941219 41363149

[phy271130-bib-0005] Bakari, G. , Mollel, E. , Max, R. , & Muhairwa, A. (2018). Evaluation of stress hormone (cortisol) levels and some biochemical parameters of pigs kept under intensive Management Systems in Morogoro, Tanzania. Journal of Biology and Life Science, 9, 78.

[phy271130-bib-0006] Blache, D. , Kho, E. A. , Tilbrook, A. J. , Tomas, K. , Plush, K. , D'Souza, D. , Maloney, S. K. , & Cozzolino, D. (2024). Near or mid‐infra‐red spectroscopy of the prefrontal cortex to identify previous stressful experience in an animal. Scientific Reports, 14, 27594. 10.1038/s41598-024-79171-y PMC11555064 39528591

[phy271130-bib-0007] Bond, C. S. , & Fox, A. H. (2009). Paraspeckles: Nuclear bodies built on long noncoding RNA. The Journal of Cell Biology, 186, 637–644. 10.1083/jcb.200906113 PMC2742191 19720872

[phy271130-bib-0008] Bu, F. , Wang, A. , Zhu, Y. , You, H. M. , Zhang, Y. F. , Meng, X. M. , Huang, C. , & Li, J. (2020). LncRNA NEAT1: Shedding light on mechanisms and opportunities in liver diseases. Liver International, 40, 2612–2626. 10.1111/liv.14629 32745314

[phy271130-bib-0009] Butler, A. A. , Johnston, D. R. , Kaur, S. , & Lubin, F. D. (2019). Long noncoding RNA NEAT1 mediates neuronal histone methylation and age‐related memory impairment. Science Signaling, 12, eaaw9277. 10.1126/scisignal.aaw9277 PMC7219525 31266852

[phy271130-bib-0010] Chen, L.‐L. , & Carmichael, G. G. (2009). Altered nuclear retention of mRNAs containing inverted repeats in human embryonic stem cells: Functional role of a nuclear noncoding RNA. Molecular Cell, 35, 467–478. 10.1016/j.molcel.2009.06.027 PMC2749223 19716791

[phy271130-bib-0011] Cheng, C. , Spengler, R. M. , Keiser, M. S. , Monteys, A. M. , Rieders, J. M. , Ramachandran, S. , & Davidson, B. L. (2018). The long non‐coding RNA NEAT1 is elevated in polyglutamine repeat expansion diseases and protects from disease gene‐dependent toxicities. Human Molecular Genetics, 27, 4303–4314. 10.1093/hmg/ddy331 PMC6276831 30239724

[phy271130-bib-0012] Choudhry, H. , Albukhari, A. , Morotti, M. , Haider, S. , Moralli, D. , Smythies, J. , Schödel, J. , Green, C. M. , Camps, C. , Buffa, F. , Ratcliffe, P. , Ragoussis, J. , Harris, A. L. , & Mole, D. R. (2015). Tumor hypoxia induces nuclear paraspeckle formation through HIF‐2α dependent transcriptional activation of NEAT1 leading to cancer cell survival. Oncogene, 34, 4482–4490. 10.1038/onc.2014.378 PMC4430310 25417700

[phy271130-bib-0013] Cinar, M. U. , Islam, M. A. , Pröll, M. , Kocamis, H. , Tholen, E. , Tesfaye, D. , Looft, C. , Schellander, K. , & Uddin, M. J. (2013). Evaluation of suitable reference genes for gene expression studies in porcine PBMCs in response to LPS and LTA. BMC Research Notes, 6, 1–14. 10.1186/1756-0500-6-56 PMC3584940 23394600

[phy271130-bib-0014] Dalgleish, T. (2004). The emotional brain. Nature Reviews. Neuroscience, 5, 583–589. 10.1038/nrn1432 15208700

[phy271130-bib-0015] Fox, A. H. , Nakagawa, S. , Hirose, T. , & Bond, C. S. (2018). Paraspeckles: Where long noncoding RNA meets phase separation. Trends in Biochemical Sciences, 43, 124–135. 10.1016/j.tibs.2017.12.001 29289458

[phy271130-bib-0016] Hemsworth, P. (2018). Key determinants of pig welfare: Implications of animal management and housing design on livestock welfare. Animal Production Science, 58, 1375–1386.

[phy271130-bib-0017] Hemsworth, P. H. , Barnett, J. L. , & Hansen, C. (1986). The influence of handling by humans on the behaviour, reproduction and corticosteroids of male and female pigs. Applied Animal Behaviour Science, 15, 303–314.

[phy271130-bib-0018] Hirose, T. , Virnicchi, G. , Tanigawa, A. , Naganuma, T. , Li, R. , Kimura, H. , Yokoi, T. , Nakagawa, S. , Bénard, M. , Fox, A. H. , & Pierron, G. (2014). NEAT1 long noncoding RNA regulates transcription via protein sequestration within subnuclear bodies. Molecular Biology of the Cell, 25, 169–183. 10.1091/mbc.E13-09-0558 PMC3873887 24173718

[phy271130-bib-0019] Hirose, T. , Yamazaki, T. , & Nakagawa, S. (2019). Molecular anatomy of the architectural NEAT1 noncoding RNA: The domains, interactors, and biogenesis pathway required to build phase‐separated nuclear paraspeckles. Wiley Interdisciplinary Reviews: RNA, 10, e1545. 10.1002/wrna.1545 31044562

[phy271130-bib-0020] Huang, S. , Qian, K. , & Zhu, Y. (2017). Diagnostic value of the lncRNA *NEAT1* in peripheral blood mononuclear cells of patients with sepsis. Disease Markers, 2017, e7962836. 10.1155/2017/7962836 PMC5804381 29463949

[phy271130-bib-0021] Ingram, H. B. , & Fox, A. H. (2024). Unveiling the intricacies of paraspeckle formation and function. Current Opinion in Cell Biology, 90, 102399. 10.1016/j.ceb.2024.102399 39033706

[phy271130-bib-0022] Irwin, A. B. , Martina, V. , Jago, S. C. S. , Bahabry, R. , Schreiber, A. M. , & Lubin, F. D. (2023). The lncRNA Neat1 is associated with astrocyte reactivity and memory deficits in a mouse model of Alzheimer's disease. bioRxiv: the Preprint Server for Biology, 2023.05.03.539260. 10.1101/2023.05.03.539260

[phy271130-bib-0023] Jensen, J. H. , Conley, L. N. , Hedegaard, J. , Nielsen, M. , Young, J. F. , Oksbjerg, N. , Hornshøj, H. , Bendixen, C. , & Thomsen, B. (2012). Gene expression profiling of porcine skeletal muscle in the early recovery phase following acute physical activity. Experimental Physiology, 97, 833–848. 10.1113/expphysiol.2011.063727 22337866

[phy271130-bib-0024] Katsel, P. , Roussos, P. , Fam, P. , Khan, S. , Tan, W. , Hirose, T. , Nakagawa, S. , Pletnikov, M. V. , & Haroutunian, V. (2019). The expression of long noncoding RNA NEAT1 is reduced in schizophrenia and modulates oligodendrocytes transcription. NPJ Schizophrenia, 5, 1–9. 10.1038/s41537-019-0071-2 PMC6386752 30696826

[phy271130-bib-0025] Kozera, B. , & Rapacz, M. (2013). Reference genes in real‐time PCR. Journal of Applied Genetics, 54, 391–406. 10.1007/s13353-013-0173-x PMC3825189 24078518

[phy271130-bib-0026] Kukharsky, M. S. , Ninkina, N. N. , An, H. , Telezhkin, V. , Wei, W. , Meritens, C. R. , Cooper‐Knock, J. , Nakagawa, S. , Hirose, T. , Buchman, V. L. , & Shelkovnikova, T. A. (2020). Long non‐coding RNA Neat1 regulates adaptive behavioural response to stress in mice. Translational Psychiatry, 10, 171. 10.1038/s41398-020-0854-2 PMC7256041 32467583

[phy271130-bib-0027] Lellahi, S. M. , Rosenlund, I. A. , Hedberg, A. , Kiær, L. T. , Mikkola, I. , Knutsen, E. , & Perander, M. (2018). The long noncoding RNA NEAT1 and nuclear paraspeckles are up‐regulated by the transcription factor HSF1 in the heat shock response. The Journal of Biological Chemistry, 293, 18965–18976. 10.1074/jbc.RA118.004473 PMC6295718 30305397

[phy271130-bib-0028] Li, R. , Harvey, A. R. , Hodgetts, S. I. , & Fox, A. H. (2017). Functional dissection of NEAT1 using genome editing reveals substantial localization of the NEAT1_1 isoform outside paraspeckles. RNA (New York, N.Y.), 23, 872–881. 10.1261/rna.059477.116 PMC5435860 28325845

[phy271130-bib-0029] Liu, C. , Gao, X. , Li, Y. , Sun, W. , Xu, Y. , Tan, Y. , du, R. , Zhong, G. , Zhao, D. , Liu, Z. , Jin, X. , Zhao, Y. , Wang, Y. , Yuan, X. , Pan, J. , Yuan, G. , Li, Y. , Xing, W. , Kan, G. , … Li, Y. (2022). The mechanosensitive lncRNA Neat1 promotes osteoblast function through paraspeckle‐dependent Smurf1 mRNA retention. Bone Research, 10, 18. 10.1038/s41413-022-00191-3 PMC8873336 35210394

[phy271130-bib-0030] Mellor, D. J. , & Beausoleil, N. J. (2015). Extending the ‘five domains’ model for animal welfare assessment to incorporate positive welfare states. Animal Welfare, 24, 241–253.

[phy271130-bib-0031] Merlot, E. , Mounier, A. M. , & Prunier, A. (2011). Endocrine response of gilts to various common stressors: A comparison of indicators and methods of analysis. Physiology & Behavior, 102, 259–265. 10.1016/j.physbeh.2010.11.009 21109031

[phy271130-bib-0032] Nakagawa, S. , Naganuma, T. , Shioi, G. , & Hirose, T. (2011). Paraspeckles are subpopulation‐specific nuclear bodies that are not essential in mice. The Journal of Cell Biology, 193, 31–39. 10.1083/jcb.201011110 PMC3082198 21444682

[phy271130-bib-0033] Nakagawa, S. , Yamazaki, T. , & Hirose, T. (2018). Molecular dissection of nuclear paraspeckles: Towards understanding the emerging world of the RNP milieu. Open Biology, 8, 180150. 10.1098/rsob.180150 PMC6223218 30355755

[phy271130-bib-0034] NCBI . (2023). Primer‐BLAST ‐ Primer Selction Tool.

[phy271130-bib-0035] NCBI . (2024). NCBI Blast: Nuclotide Sequence.

[phy271130-bib-0036] Pan, S. , Liu, R. , Wu, X. , Ma, K. , Luo, W. , Nie, K. , Zhang, C. , Meng, X. , Tong, T. , Chen, X. , Wang, X. , & Deng, M. (2021). LncRNA NEAT1 mediates intestinal inflammation by regulating TNFRSF1B. Annals of Translational Medicine, 9, 773. 10.21037/atm-21-34 PMC8246228 34268386

[phy271130-bib-0037] Park, M. K. , Zhang, L. , Min, K. W. , Cho, J. H. , Yeh, C. C. , Moon, H. , Hormaechea‐Agulla, D. , Mun, H. , Ko, S. , Lee, J. W. , Jathar, S. , Smith, A. S. , Yao, Y. , Giang, N. T. , Vu, H. H. , Yan, V. C. , Bridges, M. C. , Kourtidis, A. , Muller, F. , … Song, M. S. (2021). *NEAT1* is essential for metabolic changes that promote breast cancer growth and metastasis. Cell Metabolism, 33, 2380–2397.e9. 10.1016/j.cmet.2021.11.011 PMC8813003 34879239

[phy271130-bib-0038] Patterson, J. L. , Beltranena, E. , & Foxcroft, G. R. (2010). The effect of gilt age at first estrus and breeding on third estrus on sow body weight changes and long‐term reproductive performance. Journal of Animal Science, 88, 2500–2513. 10.2527/jas.2008-1756 20228234

[phy271130-bib-0039] Pfaffl, M. W. (2001). A new mathematical model for relative quantification in real‐time RT–PCR. Nucleic Acids Research, 29, e45. 10.1093/nar/29.9.e45 PMC55695 11328886

[phy271130-bib-0040] Pfaffl, M. W. , Tichopad, A. , Prgomet, C. , & Neuvians, T. P. (2004). Determination of stable housekeeping genes, differentially regulated target genes and sample integrity: BestKeeper – Excel‐based tool using pair‐wise correlations. Biotechnology Letters, 26, 509–515. 10.1023/b:bile.0000019559.84305.47 15127793

[phy271130-bib-0041] Puthiyedth, N. , Riveros, C. , Berretta, R. , & Moscato, P. (2016). Identification of differentially expressed genes through integrated study of Alzheimer's disease affected brain regions. PLoS One, 11, e0152342. 10.1371/journal.pone.0152342 PMC4822961 27050411

[phy271130-bib-0042] Rodrigues, A. C. , Adamoski, D. , Genelhould, G. , Zhen, F. , Yamaguto, G. E. , Araujo‐Souza, P. S. , Nogueira, M. B. , Raboni, S. M. , Bonatto, A. C. , Gradia, D. F. , & Carvalho de Oliveira, J. (2021). NEAT1 and MALAT1 are highly expressed in saliva and nasopharyngeal swab samples of COVID‐19 patients. Molecular Oral Microbiology, 36, 291–294. 10.1111/omi.12351 PMC8661855 34463043

[phy271130-bib-0004] Roozen, A. W. , Tsuma, V. T. , & Magnusson, U. (1995). Effects of short‐term restraint stress on plasma concentrations of catecholamines, beta‐endorphin, and cortisol in gilts. American Journal of Veterinary Research, 56, 1225–1227. 7486403

[phy271130-bib-0043] Silva, B. A. , Mattucci, C. , Krzywkowski, P. , Cuozzo, R. , Carbonari, L. , & Gross, C. T. (2016). The ventromedial hypothalamus mediates predator fear memory. The European Journal of Neuroscience, 43, 1431–1439. 10.1111/ejn.13239 PMC4899089 26991018

[phy271130-bib-0044] Silver, N. , Best, S. , Jiang, J. , & Thein, S. L. (2006). Selection of housekeeping genes for gene expression studies in human reticulocytes using real‐time PCR. BMC Molecular Biology, 7, 33. 10.1186/1471-2199-7-33 PMC1609175 17026756

[phy271130-bib-0045] Smith, N. E. , Spencer‐Merris, P. , Fox, A. H. , Petersen, J. , & Michael, M. Z. (2022). The long and the short of it: NEAT1 and cancer cell metabolism. Cancers, 14, 4388. 10.3390/cancers14184388 PMC9497099 36139550

[phy271130-bib-0046] Terry, R. , Nowland, T. L. , van Wettere, W. H. E. J. , & Plush, K. J. (2021). Synthetic olfactory agonist use in the farrowing house to reduce sow distress and improve piglet survival. Animals, 11, 2613. 10.3390/ani11092613 PMC8470681 34573580

[phy271130-bib-0047] Tong, X. , Shen, C. , Chen, R. , Gao, S. , Liu, X. , Schinckel, A. P. , & Zhou, B. (2019). Reestablishment of social hierarchies in weaned pigs after mixing. Animals: an Open Access Journal from MDPI, 10, 36. 10.3390/ani10010036 PMC7022989 31878050

[phy271130-bib-0048] Turner, A. I. , & Tilbrook, A. J. (2006). Stress, cortisol and reproduction in female pigs. Society of Reproduction and Fertility Supplement, 62, 191–203. 16866318

[phy271130-bib-0049] UCSC . (2023). Genome Browser.

[phy271130-bib-0050] Vandesompele, J. , de Preter, K. , Pattyn, F. , Poppe, B. , van Roy, N. , de Paepe, A. , & Speleman, F. (2002). Accurate normalization of real‐time quantitative RT‐PCR data by geometric averaging of multiple internal control genes. Genome Biology, 3, research0034.1. 10.1186/gb-2002-3-7-research0034 PMC126239 12184808

[phy271130-bib-0051] Vidović, A. , Vidović Juras, D. , Vučićević Boras, V. , Lukač, J. , Grubišić‐Ilić, M. , Rak, D. , & Sabioncello, A. (2012). Determination of leucocyte subsets in human saliva by flow cytometry. Archives of Oral Biology, 57, 577–583. 10.1016/j.archoralbio.2011.10.015 22118990

[phy271130-bib-0052] Wang, Q. , Liu, L. , Zhang, S. , Ming, Y. , Liu, S. , Cheng, K. , & Zhao, Y. (2020). Long noncoding RNA NEAT1 suppresses hepatocyte proliferation in fulminant hepatic failure through increased recruitment of EZH2 to the LATS2 promoter region and promotion of H3K27me3 methylation. Experimental & Molecular Medicine, 52, 461–472. 10.1038/s12276-020-0387-z PMC7156754 32157157

[phy271130-bib-0053] Wang, S. , Zuo, H. , Jin, J. , Lv, W. , Xu, Z. , Fan, Y. , Zhang, J. , & Zuo, B. (2019). Long noncoding RNA Neat1 modulates myogenesis by recruiting Ezh2. Cell Death & Disease, 10, 505. 10.1038/s41419-019-1742-7 PMC6594961 31243262

[phy271130-bib-0054] Wang, Y. , Hu, S. B. , Wang, M. R. , Yao, R. W. , Wu, D. , Yang, L. , & Chen, L. L. (2018). Genome‐wide screening of NEAT1 regulators reveals cross‐regulation between paraspeckles and mitochondria. Nature Cell Biology, 20, 1145–1158. 10.1038/s41556-018-0204-2 30250064

[phy271130-bib-0055] Wang, Z. , Zhen, W. , Wang, Q. , Sun, Y. , Jin, S. , Yu, S. , Wu, X. , Zhang, W. , Zhang, Y. , Xu, F. , Wang, R. , Xie, Y. , Sun, W. , Xu, J. , & Zhang, H. (2025). NEAT1 regulates BMSCs aging through disruption of FGF2 nuclear transport. Stem Cell Research & Therapy, 16, 30. 10.1186/s13287-025-04156-1 PMC11776329 39876006

[phy271130-bib-0056] Weghorst, F. , Torres Marcén, M. , Faridi, G. , Lee, Y. C. G. , & Cramer, K. S. (2024). Deep conservation and unexpected evolutionary history of neighboring lncRNAs MALAT1 and NEAT1. Journal of Molecular Evolution, 92, 30–41. 10.1007/s00239-023-10151-y PMC10869381 38189925

[phy271130-bib-0057] Wirthgen, E. , Kunze, M. , Goumon, S. , Walz, C. , Höflich, C. , Spitschak, M. , Brenmoehl, J. , Kanitz, E. , Tuchscherer, M. , Otten, W. , Gimsa, U. , Schön, P. , Manteuffel, C. , Tuchscherer, A. , Pfuhl, R. , Metges, C. C. , Stabenow, B. , Erdmann, S. , Schluricke, K. , … Hoeflich, A. (2017). Interference of stress with the somatotropic axis in pigs – Lights on new biomarkers. Scientific Reports, 7, 12055. 10.1038/s41598-017-11521-5 PMC5608691 28935925

[phy271130-bib-0058] Xie, F. , Xiao, P. , Chen, D. , Xu, L. , & Zhang, B. (2012). miRDeepFinder: A miRNA analysis tool for deep sequencing of plant small RNAs. Plant Molecular Biology, 80, 75–84. 10.1007/s11103-012-9885-2 22290409

[phy271130-bib-0059] Yu, X. , Li, Z. , Zheng, H. , Chan, M. T. V. , & Wu, W. K. K. (2017). NEAT1: A novel cancer‐related long non‐coding RNA. Cell Proliferation, 50, e12329. 10.1111/cpr.12329 PMC6529067 28105699

[phy271130-bib-0060] Zhang, H. , Xu, R. , Li, B. , Xin, Z. , Ling, Z. , Zhu, W. , Li, X. , Zhang, P. , Fu, Y. , Chen, J. , Liu, L. , Cheng, J. , & Jiang, H. (2022). LncRNA NEAT1 controls the lineage fates of BMSCs during skeletal aging by impairing mitochondrial function and pluripotency maintenance. Cell Death and Differentiation, 29, 351–365. 10.1038/s41418-021-00858-0 PMC8816946 34497381

