# Supplementary material for: Expression of the long noncoding RNA Neat1 in saliva, blood, and ventromedial hypothalamus of pigs exposed to psychosocial stressors
Source: Physiol Rep. 2026 Oct 20;14(20):e71130. doi: 10.14814/phy2.71130 (PMC13681019; doi:10.14814/phy2.71130)
Supplement: Supplementary file 1 [file PHY2-14-e71130-s001.docx]

# Supplementary

Pig ventromedial hypothalamus RNA yields

| ID | Sex | Acute | Chronic | Sample Mass (mg) | yield (μg) | 260/280 | 260/230 |
| --- | --- | --- | --- | --- | --- | --- | --- |
| 60560 | M | N | Control | 81 | 19.462 | 1.97 | 1.6 |
| 60555 | M | Y | Positive | 82 | 50.369 | 1.88 | 1.65 |
| 60553 | M | N | Control | 65 | 33.929 | 2.03 | 2.16 |
| 60552 | M | Y | Negative | 46 | 28.3635 | 1.98 | 2.2 |
| 60550 | M | N | Positive | 36 | 22.533 | 2.01 | 2.19 |
| 60544 | M | Y | Positive | 61 | 38.568 | 2.06 | 2.2 |
| 60537 | M | N | Negative | 62 | 46.025 | 2.01 | 2.13 |
| 60535 | M | N | Negative | 47 | 19.92 | 1.99 | 1.75 |
| 60534 | M | Y | Positive | 100 | 60.816 | 2.01 | 2.01 |
| 60528 | M | Y | Control | 66 | 29.834 | 1.91 | 2.11 |
| 60527 | M | N | Negative | 92 | 70.735 | 2.02 | 2.09 |
| 60522 | M | Y | Negative | 48 | 27.2475 | 2.01 | 2.19 |
| 60513 | M | N | Positive | 63 | 34.4855 | 2.04 | 2.23 |
| 60508 | M | N | Control | 43 | 37.6965 | 2 | 2.11 |
| 60503 | M | Y | Negative | 67 | 24.984 | 1.93 | 2.08 |
| 60502 | M | N | Positive | 113 | 71.49 | 2.01 | 2.05 |
| 60494 | M | Y | Control | 60 | 36.729 | 2.06 | 2.16 |
| 60488 | F | Y | Negative | 55 | 30.5515 | 2.04 | 2.15 |
| 60487 | F | Y | Control | 60 | 41.4 | 2.03 | 2.23 |
| 60484 | F | N | Negative | 48 | 24.556 | 2.05 | 2.18 |
| 60471 | F | Y | Negative | 81 | 42.705 | 1.99 | 2.16 |
| 60467 | F | Y | Positive | 61 | 13.06 | 2.19 | 2.16 |
| 60464 | F | N | Negative | 73 | 26.403 | 2.01 | 2.17 |
| 60461 | F | Y | Positive | 60 | 42.336 | 2.01 | 2.08 |
| 60456 | F | N | Negative | 70 | 2.556 | 1.83 | 2.01 |
| 60454 | F | N | Control | 45 | 31.1815 | 2.01 | 1.85 |
| 60453 | F | Y | Negative | 62 | 35.1575 | 2 | 1.96 |
| 60446 | F | Y | Control | 70 | 47.3 | 2.02 | 2.15 |
| 60444 | F | N | Control | 62 | 35.42 | 1.98 | 2.07 |
| 60441 | F | N | Control | 81 | 28.206 | 1.96 | 2.15 |
| 60436 | F | N | Positive | 60 | 37.8495 | 2 | 1.88 |
| 60434 | F | N | Positive | 58 | 18.942 | 1.91 | 1.34 |
| 60430 | F | Y | Positive | 53 | 45.18 | 2.03 | 2.16 |
| 60426 | F | N | Positive | 68 | 26.516 | 1.97 | 2.12 |
| 40463 | F | Y | Control | 48 | 22.73 | 2.01 | 2.11 |
| 20700 | M | Y | Control | 46 | 31.806 | 1.98 | 2.2 |

Blood RNA yields (before)

| ID | Sex | Acute | Chronic | Sample Volume (ul) | yield (ug) | 260/280 | 260/230 |
| --- | --- | --- | --- | --- | --- | --- | --- |
| 60560 | M | N | Control | 400 | 4.2805 | 2.08 | 1.94 |
| 60555 | M | Y | Positive | 400 | 7.35 | 2.07 | 1.99 |
| 60553 | M | N | Control | 400 | 10.241 | 2.06 | 2.01 |
| 60552 | M | Y | Negative | 400 | 11.844 | 2.07 | 2.04 |
| 60550 | M | N | Positive | 400 | 6.6675 | 2.07 | 2 |
| 60544 | M | Y | Positive | 400 | 4.7565 | 2.05 | 1.89 |
| 60537 | M | N | Negative | 400 | 6.7795 | 2.04 | 2.07 |
| 60535 | M | N | Negative | 400 | 5.3445 | 2.05 | 1.93 |
| 60534 | M | Y | Positive | 400 | 8.3265 | 2.05 | 2 |
| 60528 | M | Y | Control | 400 | 1.925 | 2.03 | 1.54 |
| 60527 | M | N | Negative | 400 | 5.0295 | 2.06 | 1.9 |
| 60522 | M | Y | Negative | 400 | 8.9915 | 2.07 | 2.13 |
| 60513 | M | N | Positive | 400 | 6.2335 | 2.07 | 2 |
| 60508 | M | N | Control | 400 | 8.715 | 2.08 | 2.03 |
| 60503 | M | Y | Negative | 400 | 5.425 | 2.06 | 1.89 |
| 60502 | M | N | Positive | 400 | 6.1985 | 2.07 | 2.01 |
| 60494 | M | Y | Control | 400 | 8.2425 | 2.09 | 2.11 |
| 60488 | F | Y | Negative | 400 | 8.4 | 2.07 | 2.06 |
| 60487 | F | Y | Control | 400 | 8.624 | 2.06 | 2.1 |
| 60484 | F | N | Negative | 400 | 5.439 | 2.01 | 1.97 |
| 60471 | F | Y | Negative | 400 | 4.074 | 2.07 | 1.96 |
| 60467 | F | Y | Positive | 400 | 9.9785 | 2.04 | 2.07 |
| 60464 | F | N | Negative | 400 | 7.728 | 2.07 | 2.08 |
| 60461 | F | Y | Positive | 400 | 10.6155 | 2.07 | 2.08 |
| 60456 | F | N | Negative | 400 | 8.5575 | 2.08 | 2.04 |
| 60454 | F | N | Control | 400 | 5.6595 | 2.08 | 2.01 |
| 60453 | F | Y | Negative | 400 | 2.422 | 2.03 | 1.69 |
| 60446 | F | Y | Control | 400 | 3.143 | 2.13 | 1.86 |
| 60444 | F | N | Control | 400 | 1.6695 | 2.06 | 1.54 |
| 60441 | F | N | Control | 400 | 5.8475 | 2.08 | 2.21 |
| 60436 | F | N | Positive | 400 | 3.2445 | 2.1 | 1.86 |
| 60434 | F | N | Positive | 400 | 6.5555 | 2.05 | 1.97 |
| 60430 | F | Y | Positive | 400 | 5.383 | 2.11 | 1.98 |
| 60426 | F | N | Positive | 400 | 5.523 | 2.09 | 2.02 |
| 40463 | F | Y | Control | 400 | 5.495 | 2.09 | 1.96 |
| 20700 | M | Y | Control | 400 | 4.634 | 2.07 | 1.88 |

Blood RNA yields (after)

| ID | Sex | Acute | Chronic | Sample Volume (ul) | yield (ug) | 260/280 | 260/230 |
| --- | --- | --- | --- | --- | --- | --- | --- |
| 60560 | M | N | Control | 400 | 5.436 | 2.07 | 1.7 |
| 60555 | M | Y | Positive | 400 | 5.01 | 2.05 | 1.94 |
| 60553 | M | N | Control | 400 | 4.29 | 2.06 | 1.59 |
| 60552 | M | Y | Negative | 400 | 7.548 | 2.06 | 1.93 |
| 60550 | M | N | Positive | 400 | 6.198 | 2 | 1.79 |
| 60544 | M | Y | Positive | 400 | 7.44 | 2.02 | 1.99 |
| 60537 | M | N | Negative | 400 | 8.34 | 2.01 | 1.91 |
| 60535 | M | N | Negative | 400 | 9.342 | 2.02 | 1.88 |
| 60534 | M | Y | Positive | 400 | 1.925 | 2.01 | 2.1 |
| 60528 | M | Y | Control | 400 | 8.58 | 2.05 | 1.87 |
| 60527 | M | N | Negative | 400 | 6.864 | 2.05 | 1.91 |
| 60522 | M | Y | Negative | 400 | 3.852 | 1.98 | 1.61 |
| 60513 | M | N | Positive | 400 | 8.202 | 2.01 | 1.81 |
| 60508 | M | N | Control | 400 | 5.388 | 2.04 | 1.76 |
| 60503 | M | Y | Negative | 400 | 4.68 | 2.1 | 1.91 |
| 60502 | M | N | Positive | 400 | 4.5 | 2.15 | 1.88 |
| 60494 | M | Y | Control | 400 | 6.684 | 2.08 | 1.85 |
| 60488 | F | Y | Negative | 400 | 4.596 | 1.93 | 1.59 |
| 60487 | F | Y | Control | 400 | 6.366 | 2.04 | 1.88 |
| 60484 | F | N | Negative | 400 | 6.612 | 2.01 | 1.65 |
| 60471 | F | Y | Negative | 400 | 6.36 | 2.06 | 1.32 |
| 60467 | F | Y | Positive | 400 | 1.795 | 2.05 | 2.12 |
| 60464 | F | N | Negative | 400 | 9.462 | 1.96 | 1.39 |
| 60461 | F | Y | Positive | 400 | 3.768 | 2.09 | 2.05 |
| 60456 | F | N | Negative | 400 | 2.88 | 2.08 | 1.64 |
| 60454 | F | N | Control | 400 | 3.33 | 2.01 | 1.65 |
| 60453 | F | Y | Negative | 400 | 4.248 | 2.06 | 1.61 |
| 60446 | F | Y | Control | 400 | 2.694 | 2.12 | 2.07 |
| 60444 | F | N | Control | 400 | 6.378 | 2.06 | 1.22 |
| 60441 | F | N | Control | 400 | 5.046 | 2.02 | 1.71 |
| 60436 | F | N | Positive | 400 | 4.11 | 2.09 | 1.62 |
| 60434 | F | N | Positive | 400 | 12.96 | 1.91 | 1.65 |
| 60430 | F | Y | Positive | 400 | 6.21 | 2.08 | 1.9 |
| 60426 | F | N | Positive | 400 | 6.318 | 2.03 | 1.71 |
| 40463 | F | Y | Control | 400 | 4.95 | 2.06 | 1.91 |
| 20700 | M | Y | Control | 400 | 7.29 | 1.99 | 1.85 |

Saliva RNA yields (after)

| ID | Sex | Acute | Chronic | yield (ug) | 260/280 | 260/230 |
| --- | --- | --- | --- | --- | --- | --- |
| 60560 | M | N | Control | 11.07 | 2.03 | 1.68 |
| 60555 | M | Y | Positive | 2.99 | 1.93 | 1.93 |
| 60553 | M | N | Control | 3.32 | 2.05 | 1.55 |
| 60552 | M | Y | Negative | 2.93 | 2.02 | 1.46 |
| 60550 | M | N | Positive | 2.15 | 2.04 | 1.36 |
| 60544 | M | Y | Positive | 0.72 | 1.77 | 1.25 |
| 60537 | M | N | Negative | 6.22 | 2.08 | 1.83 |
| 60535 | M | N | Negative | 6.92 | 2.08 | 2.05 |
| 60534 | M | Y | Positive | 1.39 | 1.97 | 0.56 |
| 60528 | M | Y | Control | 1.95 | 1.95 | 1.26 |
| 60527 | M | N | Negative | 2.06 | 2.07 | 1.46 |
| 60522 | M | Y | Negative | 2.56 | 1.99 | 1.57 |
| 60513 | M | N | Positive | 2.36 | 2.03 | 1.97 |
| 60508 | M | N | Control | 1.28 | 1.98 | 1.3 |
| 60503 | M | Y | Negative | 1.24 | 2.07 | 1.23 |
| 60502 | M | N | Positive | 1.67 | 2.02 | 1.46 |
| 60494 | M | Y | Control | 1.63 | 1.98 | 1.24 |
| 60488 | F | Y | Negative | 1.38 | 1.74 | 0.8 |
| 60487 | F | Y | Control | 1.93 | 2.04 | 1.48 |
| 60484 | F | N | Negative | 3.31 | 2.04 | 1.66 |
| 60471 | F | Y | Negative | 5.85 | 2.09 | 1.61 |
| 60467 | F | Y | Positive | 1.23 | 1.96 | 1.41 |
| 60464 | F | N | Negative | 6.50 | 2.05 | 1.65 |
| 60461 | F | Y | Positive | 1.80 | 2.05 | 1.61 |
| 60456 | F | N | Negative | 1.34 | 1.94 | 1.27 |
| 60454 | F | N | Control | 1.73 | 1.75 | 0.87 |
| 60453 | F | Y | Negative | 1.93 | 1.89 | 1.43 |
| 60446 | F | Y | Control | 4.03 | 2.02 | 1.39 |
| 60444 | F | N | Control | 1.16 | 1.98 | 1.07 |
| 60441 | F | N | Control | 1.18 | 2.01 | 1.05 |
| 60436 | F | N | Positive | 1.38 | 1.85 | 0.74 |
| 60434 | F | N | Positive | 1.95 | 1.85 | 1.01 |
| 60430 | F | Y | Positive | 0.92 | 2.02 | 1.01 |
| 60426 | F | N | Positive | 3.49 | 2.05 | 1.57 |
| 40463 | F | Y | Control | 2.44 | 2.01 | 1.49 |
| 20700 | M | Y | Control | 4.94 | 2.07 | 1.55 |
